# Repurposing of the Antiepileptic Drug Levetiracetam to Restrain Neuroendocrine Prostate Cancer and Inhibit Mast Cell Support to Adenocarcinoma

**DOI:** 10.3389/fimmu.2021.622001

**Published:** 2021-03-02

**Authors:** Roberta Sulsenti, Barbara Frossi, Lucia Bongiovanni, Valeria Cancila, Paola Ostano, Irene Fischetti, Claudia Enriquez, Francesca Guana, Giovanna Chiorino, Claudio Tripodo, Carlo E. Pucillo, Mario P. Colombo, Elena Jachetti

**Affiliations:** ^1^Molecular Immunology Unit, Department of Research, Fondazione Istituto di Ricerca e Cura a Carattere Scientifico (IRCCS) Istituto Nazionale dei Tumori, Milan, Italy; ^2^Immunology Section, Department of Medicine, University of Udine, Udine, Italy; ^3^Tumor Immunology Unit, Department of Health Sciences, University of Palermo, Palermo, Italy; ^4^Laboratory of Cancer Genomics, Fondazione Edo ed Elvo Tempia, Biella, Italy

**Keywords:** prostate cancer, neuroendocrine differentiation, mast cells, drug repurposing, tumor microenvironment, mouse models

## Abstract

A relevant fraction of castration-resistant prostate cancers (CRPC) evolve into fatal neuroendocrine (NEPC) tumors in resistance to androgen deprivation and/or inhibitors of androgen receptor pathway. Therefore, effective drugs against both CRPC and NEPC are needed. We have previously described a dual role of mast cells (MCs) in prostate cancer, being capable to promote adenocarcinoma but also to restrain NEPC. This finding suggests that a molecule targeting both MCs and NEPC cells could be effective against prostate cancer. Using an *in silico* drug repurposing approach, here we identify the antiepileptic drug levetiracetam as a potential candidate for this purpose. We found that the protein target of levetiracetam, SV2A, is highly expressed by both NEPC cells and MCs infiltrating prostate adenocarcinoma, while it is low or negligible in adenocarcinoma cells. *In vitro*, levetiracetam inhibited the proliferation of NEPC cells and the degranulation of MCs. In mice bearing subcutaneous tumors levetiracetam was partially active on both NEPC and adenocarcinoma, the latter effect due to the inhibition of MMP9 release by MCs. Notably, in TRansgenic Adenocarcinoma of the Mouse Prostate (TRAMP) mice subjected to surgical castration to mimic androgen deprivation therapy, levetiracetam reduced onset and frequency of both high grade prostatic intraepithelial neoplasia, adenocarcinoma and NEPC, thus increasing the number of cured mice showing only signs of tumor regression. Our results demonstrate that levetiracetam can directly restrain NEPC development after androgen deprivation, and that it can also block adenocarcinoma progression through the inhibition of some MCs functions. These findings open the possibility of further testing levetiracetam for the therapy of prostate cancer or of MC-mediated diseases.

## Introduction

Prostate cancer is the most commonly diagnosed malignancy worldwide and is the second leading cause of cancer death among men (1). Androgen deprivation therapy (ADT) is the treatment of choice for advanced/metastatic adenocarcinoma, but its efficacy is limited, as tumors become castration resistant (CRPC). Patients with CRPC may be treated with agents inhibiting the androgen receptor pathway (ARPI), such as the AR antagonist enzalutamide. These treatments can delay tumor progression despite the failure of ADT, but resistance eventually occurs. Mechanisms of resistance rely on cellular plasticity and neuroendocrine differentiation of adenocarcinoma (CRPC-NE or treatment-related NEPC), emerging in a relevant fraction of patients (2–5). Treatment-related NEPC is characterized by few genomic alterations, including N-Myc expression, Rb loss and p53 mutations, and by de-regulation of several epigenetic and transcription factors (2, 6). This aggressive variant of prostate cancer can very rarely occur also in untreated patients and in this case it is called *de-novo* NEPC (7). Interestingly, despite treatment-related NEPC and *de-novo* NEPC present different genomic alterations they share a common transcriptional profile (8). No definitive therapeutic options are available for NEPC, and further study of the molecular mechanisms that stimulate its emergence is necessary to identify novel targets.

Whether and how the tumor microenvironment can contribute to NEPC outgrowth is still poorly investigated. We recently demonstrated that mast cells (MCs) can favor the growth of prostate adenocarcinoma, providing MMP9 at initial stages (9) and inhibiting the anti-tumor immune response (10). On the contrary, we surprisingly found that they are also able to restrain the occurrence of NEPC through a yet undefined mechanism (9, 11).

This dual role of MCs might be considered when searching novel therapeutic options for prostate cancer. Indeed, the mere targeting of MCs function could be effective against adenocarcinoma but detrimental if not combined with drugs specific for NEPC. Here, instead, we investigate an alternative approach that considers a single drug directed to a target expressed on both MCs and NEPC cells.

Drug repurposing, or repositioning, is a strategy based on the identification of new therapeutic applications of existing drugs (12). This approach has several advantages compared to conventional drug discovery, being less time and money consuming. Indeed, as toxicity and safety studies have already been performed, this facilitates and accelerates the use of repurposed drugs in clinical trials (13). Approaches for drug repositioning, with successful examples also in prostate cancer, can be divided in knowledge-based, activity-based and *in-silico* based (14). The latter is herein applied.

## Materials and Methods

### Mice and Treatments

TRAMP mice on C57BL6/J background (C57BL/6-tgN (TRAMP)8247Ng; RRID: MGI:3795485) were kindly provided by Dr. Vincenzo Bronte (Verona University Hospital, Italy), under agreement with Dr. Norman Michael Greenberg (formerly at Fred Hutchinson Cancer Research Center, Seattle, WA, USA), maintained and screened according to (15). Surgical castration was performed in 20 weeks old TRAMP mice, under anesthesia with ketamine (100 mg/Kg; Imalgene, Boeringher Ingheilm) and xilazine (5 mg/Kg; Rompun, Bayer). Carprofene (5 mg/Kg; Norocarp, Norbrook) was given before the operation and after recovery post anesthesia. Levetiracetam (Hikma Farmaceutica, 100 mg/ml concentrate for solution for infusion) was administered in drinking water, in a dose corresponding to 60 mg/Kg day, as given in patiens (16, 17), starting 1-week post castration. Cromolyn (10 mg/kg; Sigma Aldrich, Milan, Italy) was diluted in saline solution and injected intraperitoneum (i.p.) for 5 days/week. All transgenic mice were sacrificed at 30 weeks of age. For subcutaneous tumor challenge, T1525, T23, or ST4787 cells (respectively, 2 × 10^6^, 2 × 10^6^, or 1 × 10^6^ cell/mouse) were injected in the right flank of C57BL/6 male mice. Mice were monitored twice a week and sacrificed when tumor diameters reached 10 mm × 10 mm. Levetiracetam and cromolyn were administered as described above, starting from day 4 post tumor challenge. Animal housing and experimentation were approved by the Italian Ministry of Health and performed in accordance to Italian law (D.lgs 26/2014-approval number 515/2016-PR).

### Cell Lines and *in vitro* Experiments

T1525 and T23 prostate adenocarcinoma cell lines were isolated from TRAMP mice as described (9). ST4787 were isolated from a NEPC spontaneously arisen in a TRAMP mouse genetically deficient for the protein SPARC. One prostate lobe was fixed in formalin for histopathological evaluation, whereas the remaining tissue was digested with 1 mg/ml collagenase I (Gibco) for 2h at 37°C. Collected cells were plated to establish the ST4787 cell line. ST4787 cells and tumors generated by their subcutaneous injection in C57BL/6 syngeneic mice maintain the NEPC phenotype, as shown in Supplementary Figure 1. All cells were cultured in DMEM (Gibco) supplemented with 10% of fetal bovine serum (FBS; Gibco), 200 U/mL penicillin (Cambrex), 150 U/mL streptomycin, 10 mmol/L Hepes, 10 mmol/L sodium pyruvate (Gibco) and 2 mmol/L L-glutamine. All cell lines were routinely tested for Mycoplasma using the MicoAlert Mycoplasma Detection Kit (Lonza, cat no. LT07-118). No other authentication method was performed.

### Bone Marrow-Derived MCs (BMMCs) Differentiation

Bone marrow–derived MCs (BMMCs) were obtained by *in vitro* differentiation of bone marrow cells taken from mouse femurs and tibias, in RPMI (Gibco) with 10% FBS, 200 U/mL penicillin (Cambrex), 150 U/mL streptomycin, 10 mmol/L Hepes, 10 mmol/L sodium pyruvate (Gibco), and 2 mmol/L L-glutamine and 5 mmol/L beta-mercaptoethanol (Gibco), in the presence of IL-3 (20 ng/ml; Peprotech, cat. no. AF-213-13). After 5 weeks of culture, BMMCs were monitored for FceRI and c-Kit expression by flow cytometry and used if purity was more than 95%. Antibodies used were FITC-conjugated hamster anti-mouse FcεRI monoclonal antibody (Biolegend, clone MAR-1, cat. no. 134305, RRID: AB_1626102) and PE/Cy7-conjugated rat anti-mouse CD117 (c-kit) monoclonal antibody (Biolegend, clone 2B8, cat. no. 105813; RRID: AB_313222.

### BMMCs Activation, Degranulation Assays, Leukotrienes, and Cytokine Secretion

For functional *in vitro* assays, 2 × 10^6^/ml BMMCs were either left untreated (Ctr -) or stimulated for 24 h with 1 μg/ml LPS or 1 μg/ml ionomycin (Iono, Sigma). For activation with IgE and specific antigen (IgE/Ag), 1 × 10^6^/ml BMMCs were previously sensitized in complete RPMI medium for 3 h with 1 μg/ml of dinitrophenol (DNP)-specific IgE, washed twice, and then challenged 1 h with 100 ng/ml DNP (Sigma-Aldrich).

BMMCs degranulation response was determined as the percentage of beta-hexosaminidase released. 0.5 × 10^6^ BMMCs were challenged in Tyrode's buffer (130 mM NaCl, 10 mM HEPES buffer [pH 7.4], 5.6 mM glucose, 5 mM KCl, 1.4 mM CaCl_2_, 1 mM MgCl_2_ and 0.1% bovine serum albumin) with the indicated stimuli. The enzymatic activity of the released beta-hexosaminidase was assessed by the cleavage of its synthetic substrate (p-nitrophenyl N-acetyl-glucosamide, Sigma Aldrich) in p-nitrophenol and measuring the p-nitrophenol absorbance at 405 nm with a plate spectrophotometer. Results are expressed as the percentage of beta-hexosaminidase released over beta-hexosaminidase retained in the cytoplasm.

Leukotrienes C_4_, D_4_, and E_4_ were measured using a specific detection kit (Leukotriene C4/D4/E4, Biotrak™ EIA System, cat. no. RPN224, Cytivia formerly GE Healthcare Life Sciences) according to manufacturer's instructions.

Cytokine release was assessed by ELISA on cells supernatants, with specific kits (all from Invitrogen by Thermo Fisher Scientific; RRID:SCR_008452) for IL-6 (cat. no. 88-7064), TNF- α (cat. no. 88-7324), and MMP9 (cat. no. EMMMP9) following the manufacturer's instructions.

### *In vitro* Cell Proliferation

For proliferation experiments, cells were plated in 96-well-plates (5,000 cells/well) in presence of different concentrations of levetiracetam (0, 1, 10, 25, or 50 mmol/L). After 48 h, cell proliferation was assessed using the XTT cell-viability kit (Applichem, Germany, cat. no. A8088) according to the manufacturer's protocol.

### Cytospin

Tumor cells or MCs were suspended at 10^5^ cells/100 ml. Glass slides were mounted with paper pads and cuvettes with a metal holder, loaded with 100 ml of cell suspension and then centrifuged 2 min at 2,000 rpm with a cytocentrifuge. After detaching of cuvettes and filters, slides were dried overnight and then fixed for 20 min with PBS containing 2% paraformaldehyde (PFA). For immunofluorescence, after permeabilization for 10 min with PBS containing 0.5% Saponin (Sigma) and blocking with PBS containing 5% bovine serum albumin (BSA; Sigma) we followed the protocol described for paraffin-embedded tumor sections.

### Classification of Tumor Lesions in Castrated Mice

Murine urogenital apparata were fixed in formalin and embedded in paraffin. Sections (5 μM) were de-paraffined and re-hydrated, stained with H&E (BioOptica) and evaluated by a pathologist. In serial sections, immunofluorescence for luminal (CK8) and neuroendocrine (SYP) markers was performed. Please refer to the next section for specific details on immunofluorescence protocol. Murine prostate lesions were scored according to histopathological and immunophenotypical analyses as follows. Lesions defined as high-grade prostatic intraepithelial neoplasia (HGPIN) or initial adenocarcinoma (ADENO) were characterized by CK8 positive atypical cells forming distorted/ill-defined glands within the stroma. NEPC was composed of sheets and nests of medium-sized to large cells with high nuclear to cytoplasmic ratio and/or anaplastic morphology. Cells were immunoreactive for SYP and either negative (in case of pure small-cell NE carcinoma) or positive for CK8 [in case of tumors with mixed adenocarcinoma and NE features, ref. (5)]. Regression (REG) was marked by a variable degree of glandular distortion characterized by dilated lumina with flattened or focally hyperplastic epithelia in the absence of overt nuclear atypia.

### Immunohistochemistry and Immunofluorescence

Tumor samples were fixed in formalin and embedded in paraffin. Sections (5 μM) were de-paraffined and re-hydrated. Unless alternatively specified, antigen retrieval was performed utilizing the Novocastra Epitope Retrival Solution pH9 (Novocastra, Leica Biosystems), at 98°C for 30 min. For immunohistochemistry, sections were then brought to room temperature and washed with PBS. After neutralization of the endogenous peroxidase with 3% H_2_O_2_ and Fc blocking by a specific protein block, staining was revealed using a polymer detection kit (Novocastra, Leica Biosystems) or HRP-conjugated donkey anti-rabbit IgG specific secondary antibody (GE Healthcare, cat. no. RPN1004V, RRID: AB_1062582, dilution 1:300) and AEC (3-Amino-9-ethylcarbazole) as chromogenic substrate, followed by counterstaining with Harris hematoxylin (Novocastra, Leica Biosystems). Primary antibodies were: rabbit anti mouse/human SV2A polyclonal antibody (Abcam cat. no. 32942; RRID: AB_778192; dilution 1:200) and rabbit anti mouse/human MMP9 polyclonal antibody (Abcam cat. no. ab38898; RRID: AB_776512; dilution 1:500). Slides were acquired with a Leica DM4 B microscope equipped with a Leica DFC450 C digital camera, utilizing the LAS 4.8 software (Leica Biosystems). Staining intensity was quantified using the Image J software (RRID: SCR_003070). For immunofluorescence the following antibodies were used: alexa fluor 488-conjugated rabbit anti mouse/human CK8 monoclonal antibody (Abcam; clone EP1628Y, cat. no. ab192467, RRID: AB_2864346, dilution 1:200), alexa fluor 555-conjugated rabbit anti mouse SYP monoclonal antibody (Abcam; clone YE269, cat. no. ab206870, RRID: AB_2864347, dilution 1:100), unconjugated rabbit anti mouse/human SV2A polyclonal antibody (Abcam cat. no. 32942; RRID: AB_778192; dilution 1:200), unconjugated rabbit anti-mouse/human mast-cell tryptase monoclonal antibody (Abcam; cat. no. ab134932, RRID: AB_2811029; dilution 1:100), unconjugated rabbit anti-mouse/human MMP9 polyclonal antibody (Abcam cat. no. ab38898; RRID: AB_776512; dilution 1:500). Secondary antibodies were: alexa fluor 555 conjugated donkey anti-rabbit IgG (H+L) polyclonal antibody (Invitrogen; cat. no. A-31572, RRID: AB_162543; dilution 1:500); alexa fluor 488 conjugated goat anti-rabbit IgG (H+L) polyclonal antibody (Invitrogen; cat. no. A-11034, RRID: AB_2576217; dilution 1:500).

For double CK8/SYP staining after antigen retrieval sections were blocked with PBS-Tween (0.1%) containing 5% of BSA. Primary conjugated antibodies were simultaneously incubated overnight at 4°C. For double MMP9/tryptase staining, after antigen retrieval sections were blocked with PBS-Tween (0.1%) containing 5% of BSA. Primary anti-tryptase antibody was incubated 1 h at room temperature; after washing with PBS, secondary antibody was added for 30 min at room temperature. After extensively washing with PBS, we repeated the procedure with anti-MMP9 primary antibody 1 h at room temperature and secondary antibody 30 min at room temperature.

For double SV2A/tryptase staining, the Opal Multiplex IHC kit (PerkinElmer) was developed. Antigen retrieval in pH9 buffer was brought to a boil at 100% power, followed by 20% power for 15 minutes using microwave. Sections were treated with blocking buffer for 10 min at room temperature before primary antibody incubation. Slides were then incubated with Polymeric horseradish peroxidase-conjugated (HRP) secondary antibody for 10 minutes and the signal was visualized using Opal 520 fluorophore-conjugated tyramide signal amplification (TSA) at 1:50 dilution. The HRP catalyze covalent deposition of fluorophores around the marker of interest. The slides were again processed with the microwave treatment to strip primary/secondary antibody complex and allow the next antigen-antibody staining. Another round of staining was performed with the second primary antibody incubation, followed by HRP-conjugated secondary antibody and Opal 620 fluorophore-TSA conjugated at 1:50 dilution.

Staining with DAPI (ThermoFisher Scientific) was performed for 10 min at room temperature. Slides were mounted with ProLong Diamond Antifade Mountant (ThermoFisher Scientific; RRID:SCR_008452), and acquired with a Leica DM4 B microscope equipped with a Leica DFC450 C digital camera, utilizing the LAS X software (Leica Biosystems). Images were mounted using the Image J software (RRID: SCR_003070).

### Real-Time PCR

Total RNA (including both long and short RNAs) from cells was extracted using the Quick RNA micro prep kit (Zymo Research). cDNA was obtained using the MultiScribe™-Reverse Transcriptase kit (Applied Biosysyems). RT-PCR was performed in a total volume of 200 μL using the Taqman® Fast Universal PCR Master Mix (Applied Biosystems), 20 ng of cDNA and specific probes for *Ar* (Mm00442688_m1), *Syp* (Mm00436850_m1), *Ezh2* (Mm_00468464_m1), and *Gapdh* (Mm99999915_g1), all from Applied Biosystems. Values were normalized to internal control (*Gapdh*) and analyzed using the ΔCT method.

### Western Blot

5 × 10^6^ BMMCs and 2 × 10^6^ T1525, T23, and ST4787 cells were lysed in 50 μl NP-40 buffer (25 mM Tris-HCl [pH 7.4], 150 mM NaCl, 1 mM EDTA, 1% NP-40 and 5% glycerol, 1 mM Na3VO4, 50 mM NaF; Sigma Aldrich), containing protease inhibitor cocktail (Sigma, cat no.P8340) for 10 minutes on ice. Lysates were then centrifuged at 12,000 × g, 4°C for 10 min and supernatants were collected and stored at −80°C. For western blot analysis, lysates were diluted with 4 × Laemmli buffer and denatured 7 min at 95°C, separated on SDS 10% polyacrylamide gels and blotted on a nitrocellulose membrane (Amersham) at 300 V, 250 mA, 4°C for 3 h. Membranes were blocked in TBS-T (20 mM Tris-base [pH 7.6], 150 mM NaCl, 0.05% Tween-20) containing 5% BSA for 1 h at room temperature. Primary antibodies were diluted 1:500 in TBS-T containing 5% BSA and membranes probed overnight at 4°C with gentle agitation. HRP-conjugated secondary antibodies were incubated for 1 h at room temperature with gentle agitation. Signals were detected using Super Signal West Femto (Thermo Scientific cat no. 34096; RRID: SCR_008452) and acquired with a ChemiDoc imaging system (BioRad; RRID: SCR_019037). Quantification was performed with the Image J software (RRID: SCR_003070).

Primary antibodies were: rabbit anti-mouse/human SV2A polyclonal antibody (Abcam; cat. No. 32942 RRID: AB_778192, dilution 1:500) and mouse anti-mouse/human actin monoclonal antibody (Becton Dickinson; clone C4, cat. no. 612656, RRID: AB_2289199; dilution 1:1,000). Secondary antibodies HRP conjugated were: goat anti-mouse IgG (Thermo Fisher Scientific; RRID:SCR_008452; cat. no. 1858413, RRID:AB_1185566, dilution 1:2,500), and goat anti-rabbit IgG (Thermo Fisher Scientific RRID:SCR_008452; cat. no. 1858415, RRID: AB_228341, dilution 1:2,500).

### Analyses of Mouse and Human NEPC Data Sets

Raw data derived from gene expression profiling of adenocarcinoma and NEPC spontaneously occurring in TRAMP mice (18), of NEPC occurring in TRAMP mice following pharmacologic inhibition of MCs with cromolyn (9) and of TRAMP-derived prostate-cancer stem like cells with NEPC or adenocarcinoma features (19) were analyzed with the limma package available within Bioconductor (RRID: SCR_010943) (20). Class comparison was performed to retrieve NEPC-associated transcripts with log_2_ fold change >1 and with adjusted *p* < 0.05 were selected. Mouse gene symbols were converted into orthologous human gene symbols with the biomaRt package (21) and then intersected with up-regulated genes in human metastatic NEPC vs. CRPC (2). MetaCore version 19.4 (Clarivate Analytics, Philadelphia, PA, USA; RRID: SCR_008125) was used for target identification.

### Statistical Analyses

Statistical analysis was performed with the GraphPad Prism software (GraphPad Software, La Jolla, CA, USA; RRID:SCR_002798). We used Chi-Square Test for comparison of tumor frequencies in castrated mice and the Student's *T*-test, or One-Way ANOVA followed by Tukey's tests for other experiments. Values were considered statically significant for ^*^p < 0.05, ^**^p < 0.01, ^***^p < 0.001, and ^****^p < 0.0001. Number and types of replicates are indicated in each figure legend.

## Results

### *Sv2a* Is Up Regulated in Gene Expression Profiles From Murine and Human NEPC

**Figure 1:**
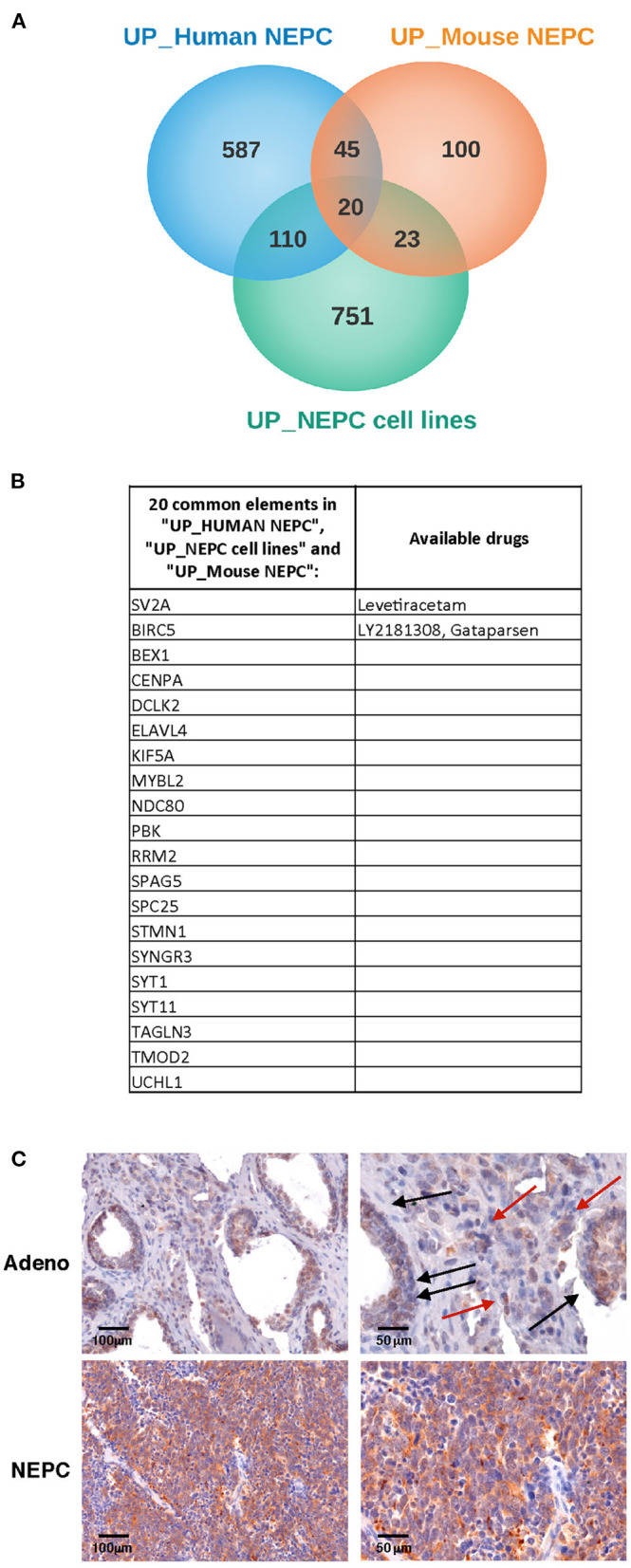
SV2A transcript and protein is overexpressed in human NEPC. **(A)** We looked for transcripts up-regulated in NEPC analyzing gene expression profiles that we previously obtained in TRAMP mice with adenocarcinoma or NEPC [“Mouse NEPC,” ref (9, 18)] and public available profiles of TRAMP-derived prostate cancer stem-like cells with NEPC or adenocarcinoma features [“NEPC cell lines,” ref (19)] and of human NEPC and CRPC patients [“human NEPC,” ref (2)]. Venn diagrams show the intersection of up-regulated genes in NEPC in the three data sets. **(B)** List of the 20 genes commonly up- regulated in mouse NEPC, NEPC cell lines and human NEPC, with indication of specific targeting drugs, when available, identified with the Metacore software. **(C)** Immunohistochemistry for SV2A (brown staining) in human prostate adenocarcinoma (upper panels; black arrows indicate positive tumor glands, red arrows indicate positive stroma cells) or NEPC (lower panels). Scale bars indicate magnifications.

Our initial aim was to identify molecules selectively expressed in NEPC. Therefore, we interrogated gene expression profiles that we have previously generated in the Transgenic Adenocarcinoma of the Mouse Prostate model [TRAMP (15)], comparing the transcriptome of adenocarcinoma and NEPC occurring in TRAMP mice either spontaneously (18) or following pharmacologic inhibition of MCs with cromolyn (9) (all identified as “Mouse NEPC”). We also included the analyses of published data by comparing the transcriptome of TRAMP-derived prostate-cancer stem-like cells with NEPC or adenocarcinoma features [(19); identified as “NEPC cell lines”]. To increase translational relevance, results obtained from murine models were compared with gene expression profiles obtained from a publicly available data set comprising human metastatic NEPC and CRPC [identified as “Human NEPC”; (2)]. In search of possible commonly expressed drug targets, we analyzed the lists of up-regulated genes (log_2_FC > 1 and adj. *p*-value < 0.05; Figure 1A). The Metacore software was used to identify drugs targeting the proteins encoded by the 20 genes commonly up regulated in all the three data sets (Figures 1A,B). We found that two of these genes, *Sv2a* and *Birc5*, encode for proteins that are targeted by available drugs (Figure 1B). *Birc5* is the gene encoding for Survivin, an antiapoptotic protein overexpressed in several cancer types and already largely investigated in clinical studies (22). Notably, in prostate cancer an antisense oligonucleotide inhibitor of Survivin (23), as well as immunotherapeutic approaches targeting this protein (24) are currently tested.

*Sv2a* encodes for the synaptic vesicle protein SV2A, a component of synaptic vesicles involved in cell exocytosis and mainly expressed in neural and endocrine cells (25). SV2A is the target of the drug levetiracetam (26), which is commonly used in epileptic patients, as it mitigates seizures (27).

To validate these results, we analyzed SV2A expression in human prostate cancer tumor samples by immunohistochemistry. According to *in silico* data, we found a dim positivity for SV2A in prostate adenocarcinoma cells and some positive infiltrating stroma cells (Figure 1C, upper panels), whereas a strong and homogeneous expression of SV2A characterized NEPC (Figure 1C, lower panels). This finding prompted us to investigate whether SV2A could be a potential therapeutic target in NEPC.

### NEPC and MCs Robustly Express SV2A

**Figure 2:**
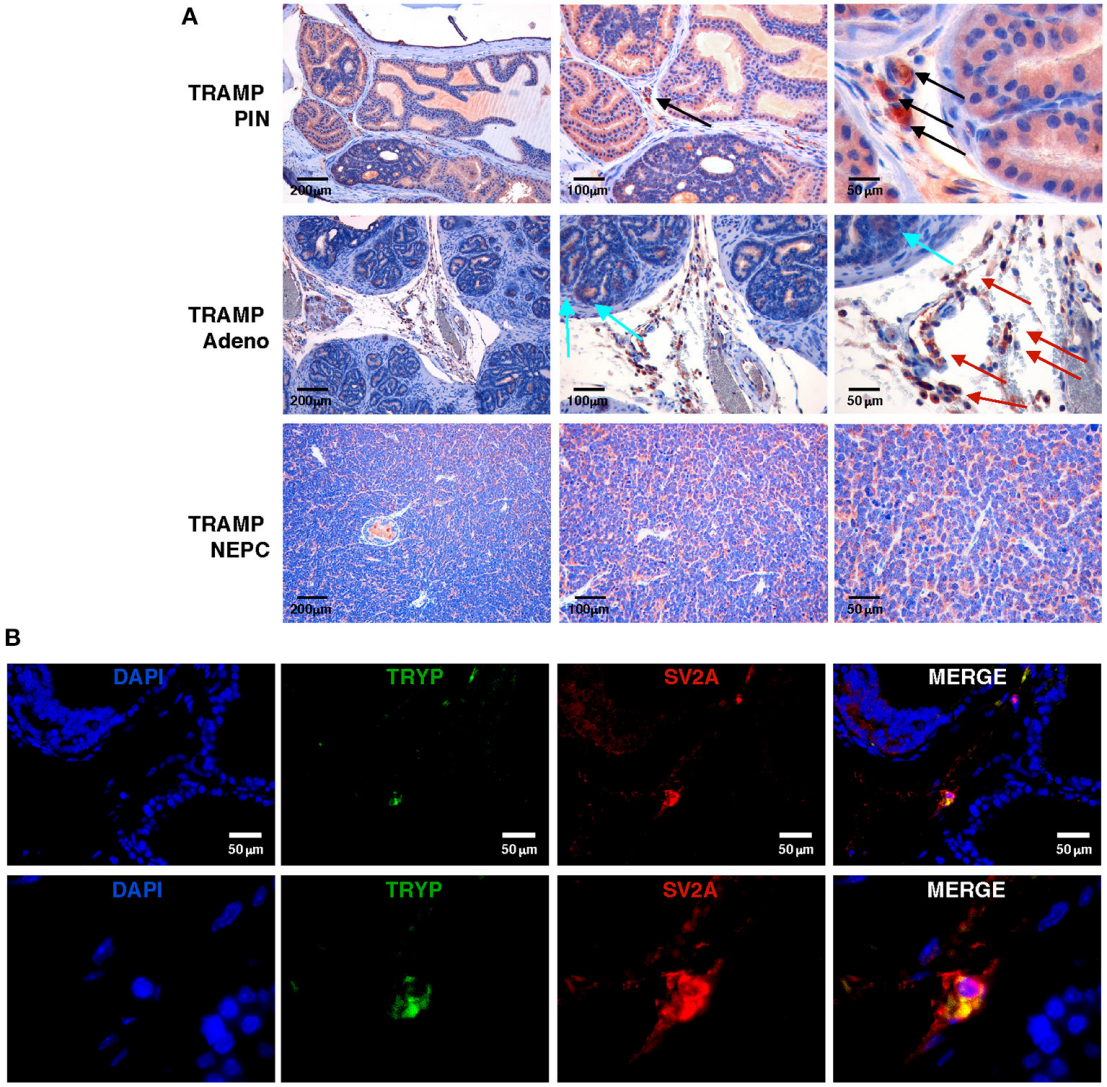
SV2A protein is expressed in murine NEPC and tumor-infiltrating MCs, *in vivo*. **(A)** Immunohistochemistry for SV2A (brown staining) on prostatic intraepithelial neoplasia (PIN), adenocarcinoma or NEPC lesions from TRAMP mice. Arrows indicate positivity in infiltrating MCs (black arrows), monocytes (red arrows) or scattered tumor cells (cyan arrows), respectively. Scale bars indicate magnifications. **(B)** Immunofluorescence for the MC-specific marker tryptase (TRYP; in green) and SV2A (in red) in TRAMP prostate with adenocarcinoma. Blue staining is DAPI. In upper panels scale bars indicate magnification. Lower panels are further digital magnifications of the original pictures.

To study the antitumor efficacy of levetiracetam, we moved to the TRAMP mouse, a preclinical prostate cancer model in which both adenocarcinoma and NEPC can occur (11, 28, 29). We investigated SV2A expression at a protein level, performing immunohistochemistry on prostate lesions from TRAMP mice. SV2A was expressed in luminal cells in prostatic intraepithelial neoplasia (PIN) whereas in adenocarcinoma lesions it was negative in almost all tumor cells with few scattered tumor cells showing mild positivity (Figure 2A upper and middle panels). According to showed *in silico* and human data (Figure 1), SV2A stained diffusely and was highly positive in all tumor cells in NEPC lesions (Figure 2A, lower panels). Interestingly, in PIN and adenocarcinoma lesions we found high expression of SV2A by infiltrating stroma cells, particularly MCs and monocytes (Figure 2A upper and middle panels). Indeed, we know from our previous work (9) that MCs infiltrate prostate lesions and that their number increases from PIN to adenocarcinoma, whereas it is low in tumors with anaplastic/NEPC features, in both TRAMP mice and patients. Besides immunohistochemistry, we also confirmed the expression of SV2A in MCs infiltrating TRAMP prostates by double immunofluorescence staining with the MC-specific marker tryptase (Figure 2B).

**Figure 3:**
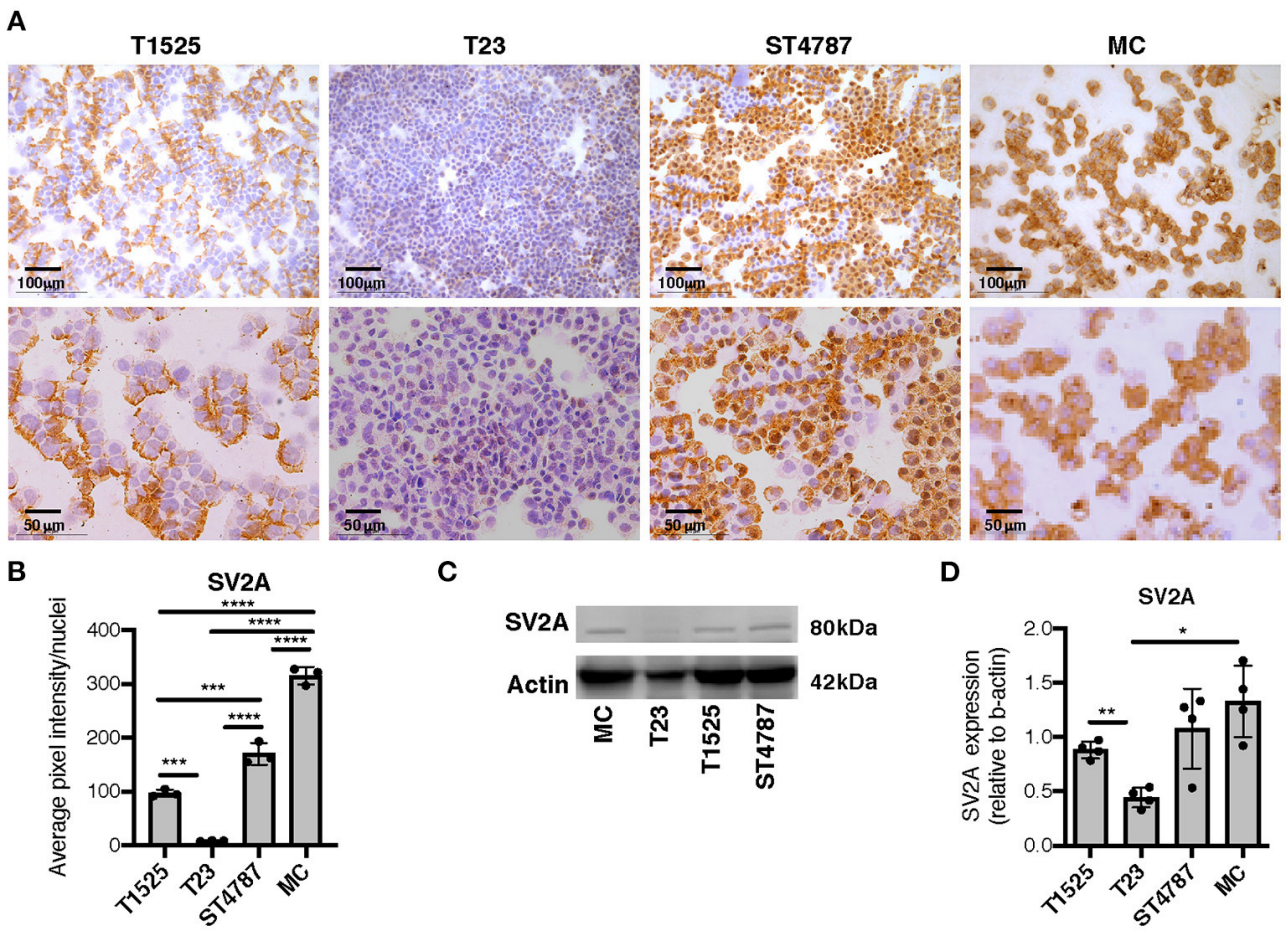
SV2A protein is expressed in murine NEPC cell lines and bone marrow derived MCs. **(A)** Immunohistochemistry for SV2A (brown staining) on cytospins of T1525, T23, or ST4787 tumor cell lines or *in vitro* cultured- bone marrow derived MCs. Scale bars indicate magnifications. **(B)** Digital quantification for SV2A staining intensity in **(B)**, performed with Image J software. Dots indicate biological replicates. **(C)** Representative western blot for SV2A on T1525, T23, ST4787 tumor cell lines and bone marrow derived MCs. Actin was used as loading control. The experiment was repeated four times. **(D)** Digital quantification of SV2A expression in **(C)**, normalized on actin, performed with Image J software. Dots indicate biological replicates. Anova followed by Tukey's test: *p < 0.05, **p < 0.01, ***p < 0.001, and ****p < 0.0001.

We then tested SV2A expression by immunohistochemistry on cytospins from a panel of TRAMP-derived prostate cancer cell lines (T1525, T23, and ST4787) generated in our laboratory [Ref (9) and Supplementary Figure 1], and on *in vitro*-cultured bone marrow-derived MCs (BMMCs). Results showed mild positivity in T1525 cells, representative of well-differentiated, initial stage adenocarcinoma, whereas T23 cells, representative of poorly differentiated adenocarcinoma post epithelial-mesenchymal transition (EMT) were negative (Figures 3A,B). We can therefore suppose that T1525 cells model those scattered adenocarcinoma cells that express mild levels of SV2A in TRAMP tumors (Figure 2A cyan arrows), whereas T23 cells are representative of the most abundant SV2A negative adenocarcinoma cells. In line with expression observed *in vivo*, both the NEPC cell line ST4787 and BMMCs were strongly positive for SV2A (Figures 3A,B). We obtained a similar pattern of SV2A expression by western blot analysis on proteins extracts from tumor cell lines and BMMCs (Figures 3C,D).

These results show that NEPC cells and MCs, both *in vitro* and *in vivo*, express high levels of SV2A, therefore suggesting that they could be both potentially affected by treatment with levetiracetam.

### Levetiracetam Slightly Reduces the Proliferation of SV2A Expressing Tumor Cells and Inhibits the Activation and Degranulation of MCs *in vitro*

**Figure 4:**
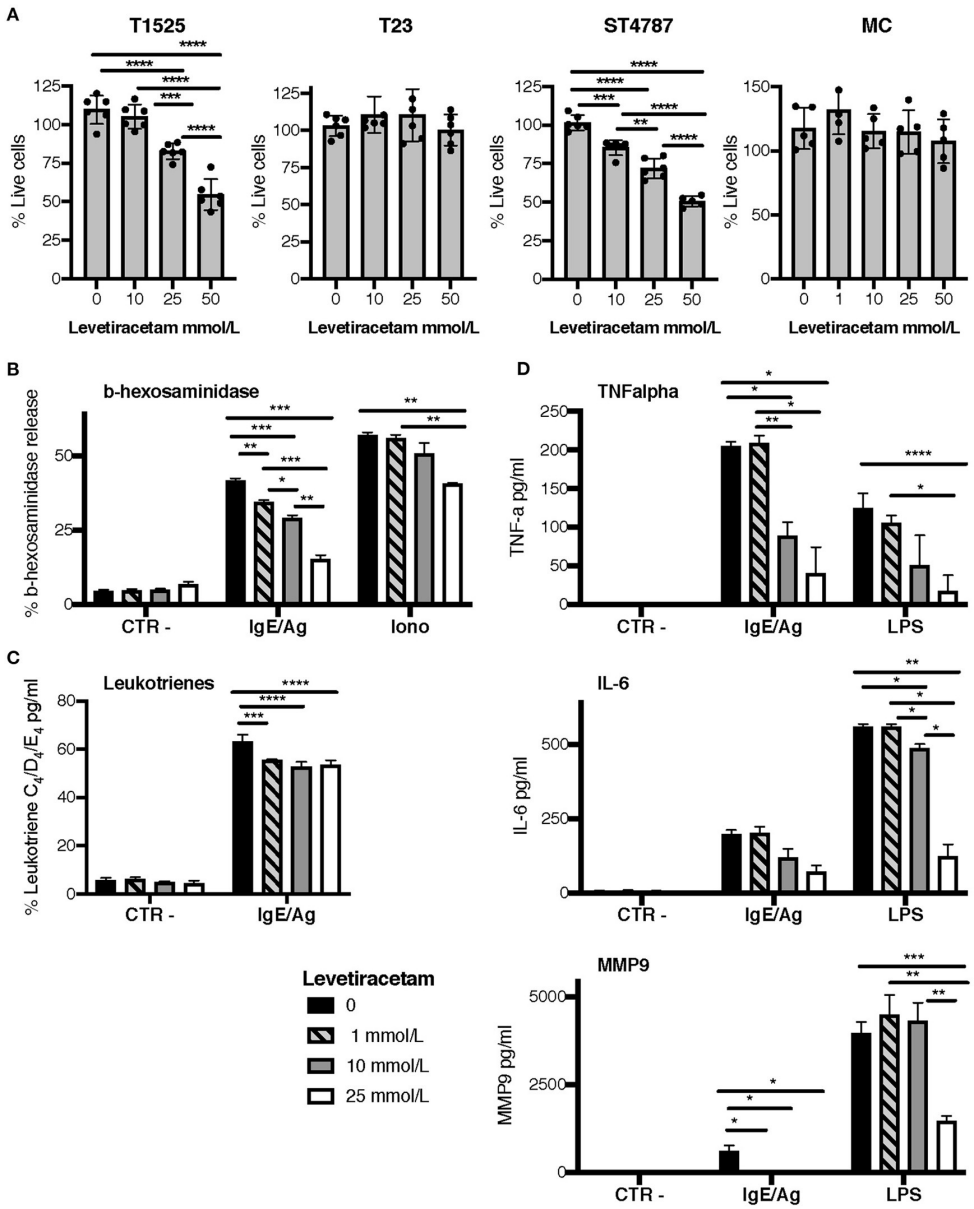
Levetiracetam reduces tumor cell proliferation and MCs degranulation. **(A)** T1525, T23, or ST4787 tumor cell lines or bone marrow derived MCs, cells were cultured for 48 h in the presence of different concentrations of levetiracetam, as indicated, and tested for proliferation with the XTT assay (Applichem). Histograms show a pool of two different experiments; dots indicate biological replicates. Anova followed by Tukey's test *p < 0.05, **p < 0.01, ***p < 0.001, and ****p < 0.0001. **(B–D)** Release of β-hexosaminidase **(B)**, leukotrienes **(C)**, and TNF-α, IL-6 and MMP9 **(D)** in bone marrow derived MCs either unstimulated (Ctr-) or stimulated with IgE/Ag, Ionomycin (Iono) or LPS. Levetiracetam was added at different concentrations, as indicated. *N* = 3 per group, experiment was repeated three times. Data were analyzed with paired two-way Anova *p < 0.05, **p < 0.01, ***p < 0.001, and ****p < 0.0001.

To verify the above hypothesis, we tested the activity of levetiracetam on prostate cancer cell lines and BMMCs after 48 h of culture. Only high concentrations of levetiracetam (25 and 50 mM) inhibited the proliferation of T1525 cells, which express mild levels of SV2A, whereas it was inactive against SV2A negative T23 cells at any of the doses tested (Figure 4A). In the NEPC cell line ST4787, expressing high levels of SV2A (Figure 3A), levetiracetam was moderately effective in reducing cell proliferation even at a lower dose (10 mM) (Figure 4A).

When tested on BMMCs, levetiracetam had no effect on cell vitality (Figure 4A). As MCs can release several soluble mediators by granule exocytosis in response to activation, we tested whether levetiracetam hampered this function in BMMCs subjected to different stimuli. Notably, this drug significantly inhibited the release of beta-hexosaminidase in both IgE/Ag- and Ionomycin-activated BMMCs, in a concentration dependent manner (Figure 4B). Moreover, levetiracetam decreased the IgE/Ag crosslink-mediated release of newly formed lipid mediators as demonstrated by the reduced amounts of leukotrienes C_4_, D_4_, and E_4_ measured in cell supernatants. Nevertheless, this effect was lower if compared to inhibition of degranulation, and it was not dose-dependent (Figure 4C). To test the possible effects of levetiracetam on modulating cytokine secretion, BMMCs were activated through the engagement of the high-affinity IgE receptor (by IgE/Ag stimulation) or of TLR4 (by LPS stimulation), and cell supernatants were tested by ELISA assays (Figure 4D). Levetiracetam reduced the quantity of IL-6 and TNF-α released by BMMCs induced by stimulation with both IgE/Ag and LPS at a drug concentration of 10 and 25 mM (Figure 4D). Interestingly, the treatment with 25 mM levetiracetam also inhibited the release of MMP9 by BMMCs in response to both stimuli (Figure 4D).

Results so far indicate that levetiracetam can reduce, albeit partially, the proliferation of SV2A expressing tumor cells, and can prevent the degranulation of MCs after stimulation.

### Levetiracetam Reduces Adenocarcinoma and NEPC *in vivo*

**Figure 5:**
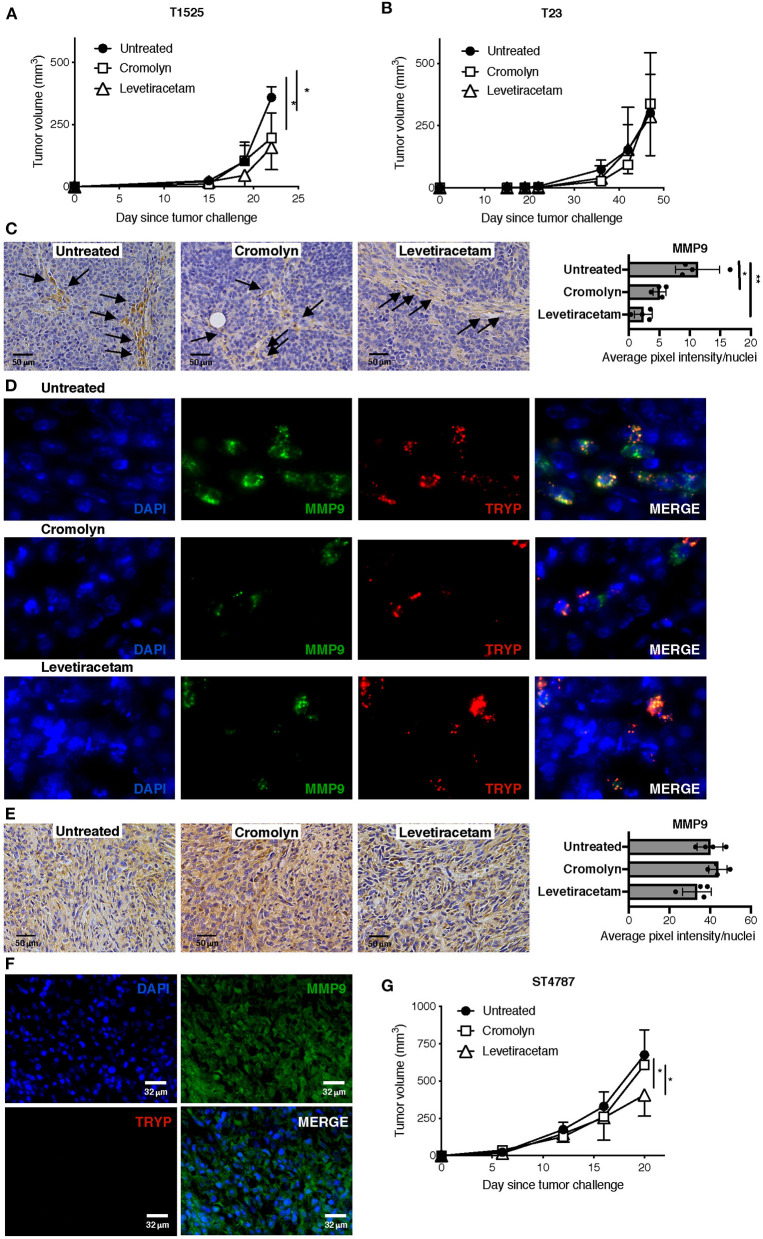
Levetiracetam partially inhibits prostate adenocarcinoma and NEPC growth in mice with subcutaneous tumors. **(A,B)** Tumor growth in C57BL/6 mice injected subcutaneously with T1525 **(A)** or T23 **(B)** cells and treated with cromolin (10 mg/Kg/Day; i.p., 5 days/week) or levetiracetam (60 mg/Kg/Day, daily in drinking water), starting from day 4 after tumor challenge. *N* = 5 mice in each group. Experiment was repeated three times. Anova followed by Tukey's test *p < 0.05. **(C)** Immunohistochemistry (brown staining) for MMP9 in T1525-derived tumors, collected from mice treated as in **(A)**. Arrows indicate MCs infiltrating the stroma. Scale bars indicate magnification. Histogram reports digital quantification of MMP9 staining intensity, performed with Image J software. Anova followed by Tukey's test *p < 0.05, **p < 0.01. **(D)** Immunofluorescence for MMP9 (in green) and the specific MC-marker tryptase (TRYP; in red) on T1525 tumors collected from untreated mice or from mice treated with cromolyn or levetiracetam as in **(A)**. Blue staining is DAPI. Images are digital magnifications of the original pictures (taken with ×630 magnification) shown in Supplementary Figure 2. **(E)** Immunohistochemistry for MMP9 in T23-derived tumors, collected from mice treated as in **(B)**. Scale bars indicate magnification. Histogram reports digital quantification of MMP9 staining intensity, performed with Image J software. **(F)** Immunofluorescence for MMP9 (in green) and the specific MC-marker tryptase (TRYP; in red) on T23 tumors collected from untreated mice. Blue staining is DAPI. Scale bars indicate magnification. **(G)**. Tumor growth in C57BL/6 mice injected subcutaneously with ST4787 cells and treated with cromolyn (10 mg/Kg/Day; i.p., 5 days/week) or levetiracetam (60 mg/Kg/Day, daily in drinking water), starting from day 4 after tumor challenge. *N* = 5 mice in each group. Experiment was repeated three times. Anova followed by Tukey's test *p < 0.05.

Given the effect of levetiracetam in inhibiting the degranulation of MCs, we investigated whether this drug could reduce their adenocarcinoma promoting function *in vivo*. We know from our previous work that MCs can sustain the growth of early-stage, well-differentiated T1525 cells when injected subcutaneously in syngeneic mice, through the provision of soluble mediators including MMP9 (9). On the contrary, MCs are dispensable for *in vivo* growth of poorly-differentiated, EMT-like T23 adenocarcinoma cells, which can self-produce MMP9 (30). We therefore compared tumor growth in mice subcutaneously challenged with T1525 or T23 cells and treated with the MC-stabilizer cromolyn or with levetiracetam. According to our hypothesis, both drugs reduced the growth of T1525-derived tumors (Figure 5A), but not of T23-derived tumors (Figure 5B). As expected, in untreated mice bearing T1525 tumors immunohistochemistry and immunofluorescence showed strong MMP9 production by infiltrating MCs and not by T1525 tumor cells (Figures 5C,D and Supplementary Figure 2), whereas in T23 tumors MMP9 was mild and diffusely expressed by tumor cells and no infiltrating MCs were detected (Figures 5E,F). Notably, treatment with either cromolyn or levetiracetam blunted MMP9 production by MCs *in vivo* (Figures 5C,D and Supplementary Figure 2), but it did not affect MMP9 expression in T23 tumor cells (Figure 5E). We can conclude that the efficacy of levetiracetam in limiting the growth of T1525-derived tumors is due, in part, to its effect in blocking MCs degranulation and consequent adenocarcinoma-promoting activity. On the contrary, levetiracetam is ineffective against T23-derived tumors, which do not express SV2A and do not depend on MCs support for their growth.

We finally tested the activity of levetiracetam against NEPC tumor cells, *in vivo*. We found that levetiracetam, but not cromolyn, partially reduced tumor growth in mice challenged with the NEPC cell line ST4787 (Figure 5G). These results indicate that, as expected from our previous data (9, 11), inhibition of MCs is ineffective against NEPC. Nevertheless, as NEPC cells express SV2A, they can be directly targeted by levetiracetam.

**Figure 6:**
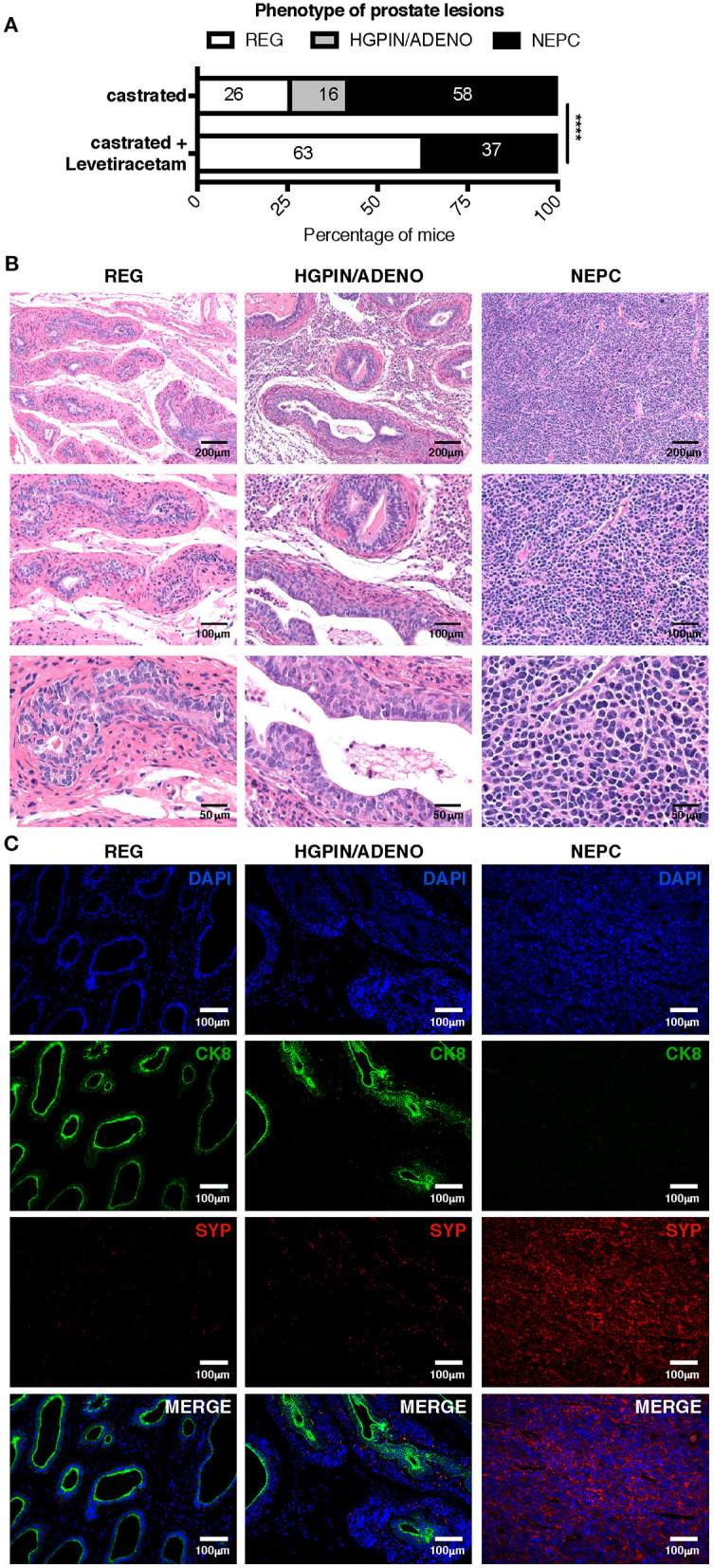
Levetiracetam inhibits adenocarcinoma and NEPC growth in castrated TRAMP mice. **(A)** TRAMP mice were castrated at 20 weeks of age and left untreated (*n* = 19) or treated daily with levetiracetam (60 mg/Kg/Day in drinking water; *n* = 19), starting one-week post castration. All mice were killed at 30 weeks of age and prostates collected for histopathology. Graph depicts the relative percentage of lesions scored as tumor involution/regression (REG), high-grade intraepithelial neoplasia with or without focal adenocarcinoma (HGPIN/ADENO) or neuroendocrine tumor (NEPC). Please refer to materials and methods for detailed criteria utilized for tumor lesion classification. Chi Square Test: ****p < 0.0001. Numbers within bar represent the relative percentage of mice presenting each type of lesion. **(B,C)** Representative images of prostates of castrated TRAMP mice showing evidence of REG, HGPIN/ADENO, or NEPC. Images show hematoxylin and eosin staining **(B)**; or immunofluorescence **(C)** staining for CK8 (green), SYP (red), and DAPI (blue). Scale bars indicate magnification.

To better mimic a clinical setting of NEPC occurring as relapse following ADT, we tested the antitumor activity of levetiracetam in TRAMP mice subjected to surgical castration. Mice were castrated at 20 weeks of age and analyzed by necropsy 10 weeks later. As from literature and our previous results, we know that untreated TRAMP mice on a C57BL/6 background spontaneously develop prostate adenocarcinoma, and *de-novo* NEPC arises in a small 10% fraction of mice (10, 28, 29). After castration, the frequency of NEPC (comprising both *de-novo* and treatment-related NEPC), raised up to 58% of mice (11 out of 19 mice; Figure 6A). Histologically, NEPC tumors were composed of sheets and nests of medium-sized to large cells with high nuclear to cytoplasmic ratio and/or anaplastic morphology (Figure 6B), expressing the NEPC marker synaptophysin (SYP) and either negative (in case of “pure” NEPC, as shown in Figure 6C) or positive for CK8 [in case of NEPC tumors with mixed adenocarcinoma and NE features, ref. (5), as shown in Supplementary Figure 1A]. The remaining mice in the castrated cohort showed signs of high-grade prostatic intraepithelial neoplasia with or without focal carcinomatous transformation characterized by CK8 positive atypical cells that formed distorted/ill-defined glands within the stroma (HGPIN/ADENO, Figures 6B,C; found in 3 out of 19, corresponding to 16% of mice; Figure 6A); or signs of glandular involution/tumor regression (REG, Figures 6B,C; found in 5 out of 19, corresponding to 26% of mice Figure 6A). Administration of levetiracetam after castration resulted in a reduction of the frequency of NEPC (found in 7 out of 19, corresponding to 37% of mice, Figure 6A) and a marked increase of mice showing only signs of regression (12 out of 19, corresponding to 63% of mice). Notably, none of the TRAMP mice treated with levetiracetam after castration developed lesions classified as HGPIN/ADENO (Figure 6A).

Altogether these findings suggest that pharmacological targeting of SV2A with the drug levetiracetam can reduce NEPC development after androgen ablation/castration. Levetiracetam can also partially inhibit the growth of prostate adenocarcinoma, via MCs inactivation.

## Discussion

Treatment with ADT/ARPI in patients with CRPC ultimately fails, due to mechanisms of resistance associated with cellular plasticity and the emergence of treatment-related NEPC (4, 6), and the diagnosis of patients with advanced prostate cancer remains dismal. Therefore, the identification of novel therapeutic targets for CRCP and NEPC is an urgent unmet clinical need.

Utilizing an *in silico* drug repurposing approach (14), we found that human and murine NEPC overexpress SV2A, which is the target of the antiepileptic drug levetiracetam. Protein-target validation by immunohistochemistry led us discover by serendipity that SV2A is expressed also by tumor-infiltrating MCs. As we previously unraveled the dual role of MCs either promoting or preventing prostate cancer according to its adenocarcinoma or NE phenotype (9, 11), the identification of SV2A as a common target on MCs and NEPC prompted us to test whether levetiracetam could be effective on both prostate cancer histotypes.

Levetiracetam has never been tested in prostate cancer cells before our study, whereas it has already showed anti-proliferative capacity against glioma cells *in vitro* (31). Albeit clinical testing of levetiracetam in patients with glioblastoma did not associate with improved progression-free survival and/or overall survival (32), a case report documented regression of lung metastatic lesions in a man with adenoid cystic carcinoma, in which levetiracetam was administered with the sole intent to relief epilepsy, and after the failure of several lines of therapy against the tumor (33). Here, we show that levetiracetam has some efficacy against both adenocarcinoma and NEPC, *in vivo*, in subcutaneous and spontaneous murine models. Nevertheless, the extent of tumor inhibition that we observed in this study is limited, but suggestive for further evaluation of levetiracetam in combination with other therapeutic approaches in prostate cancer.

*In vivo* the MC-stabilizer cromolyn and levetiracetam have comparable effect in restraining T1525-prostate cancer tumors, which strictly depend on MCs provision of MMP9 for their growth (9). Therefore, we can conclude that the activity in blocking prostate adenocarcinoma is due to the solely targeting of MCs degranulation, and exclude an additional effect of levetiracetam on adenocarcinoma cells. Indeed, albeit T1525 cells express intermediate levels of SV2A, their proliferation *in vitro* can be limited only by a very high concentration of levetiracetam, which is far higher than that of 60 mg/Kg day, used in our *in vivo* experiments and corresponding to the maximum recommended for patients (16, 17). Studies in mouse or rat models have used higher concentrations of levetiracetam up to 600 mg/Kg day without showing any additional side effect (34, 35), therefore suggesting the possibility of testing high-doses of levetiracetam *in vivo* in prostate cancer models, with the aim to target both MCs and those scattered adenocarcinoma cells expressing mild levels of SV2A.

On the contrary, the inhibitory effect of levetiracetam on NEPC is rather quite specific and depends on strong expression of SV2A in NE tumor cells. Interfering with the mechanism of cell exocytosis could indeed block the release of autocrine or paracrine factors involved in cell proliferation. According to this hypothesis, it has been shown that the botulinum toxin type A (BTA), which molecularly binds to SV2A (36), can inhibit the growth of SV2A-expressing LNCaP prostate cancer cells, both *in vitro* and *in vivo* (37).

Given the above considerations, the therapeutic efficacy of levetiracetam that we observed against both adenocarcinoma and NEPC in castrated TRAMP mice, likely results from both the inhibition of adenocarcinoma promoting-functions of MCs and from the direct action on NE tumor cells, respectively.

Additional potential antitumor activity of levetiracetam could come from the known activity as histone deacetylase (HDAC) inhibitors of several antiepileptic drugs including levetiracetam or valproic acid (38). Interestingly, a study analyzing the effect of long-term antiepileptic treatment on PSA levels in tumor-free patients, albeit limited to a low number of subjects, showed that treatment with either valproic acid or levetiracetam was associated to lower levels of circulating PSA (39).

Nonetheless, our evidence that levetiracetam does not restrain, both *in vitro* and *in vivo*, the growth of SV2A-negative T23 tumor cells, likely excludes the relevance of HDAC- inhibition properties of this drug in our setting. These data are in line with the disappointing results of clinical trials testing a variety of HDAC inhibitors in prostate cancer patients (40).

To our knowledge, besides being the first description of the use of levetiracetam against prostate cancer, it is also the first time that levetiracetam is used to inhibit the degranulation of MCs. MCs are involved in the pathogenesis of various human disorders, such as mastocytosis, urticaria, allergic rhinitis, asthma, tumors, and autoimmune diseases (41) and several MC-modulating drugs have been largely investigated in the past decades (42). These substances include molecules that reduce the number of MCs and/or inhibit the MC activation process such as omalizumab, cromolyn, and imatinib; or molecules that interfere with the process of release of mediators, including steroids and non-steroidal anti-inflammatory drugs. A variety of these MC-modulating drugs are already approved by the Food and Drug Administration Agency, especially for the treatment of hypersensitivity diseases (43). However, there are no pharmacologic agents that can exclusively and selectively suppress MC activation.

We have herein observed an inhibitory effect of levetiracetam on the release of preformed mediators as well as newly synthesized molecules from MCs that had been previously activated through different pathways. The mechanism of action of levetiracetam is not exactly clear, but it is known that this antiepileptic drug interferes with the process of exocytosis of neurotransmitters and that this exocytosis is down-regulated via reduced calcium inward currents (44). Cataldi et al. (45) demonstrated that levetiracetam inhibits calcium release from intracellular inositol-trisphosphate (IP3)-sensitive granules in PC12 cells. IP3-dependent release of calcium from intracellular stores is regarded as a major factor responsible for the consistent elevation of Ca^2+^ ions observed in epileptic neurons (46). Indeed, both the exocytosis of pre-stored granules and the release of cytokines by MCs depend on calcium signaling (47). Consequently, the impaired calcium mobilization induced by levetiracetam could decrease the magnitude of the degranulation response of MCs to different activating stimuli. This could explain the reduced amounts of released mediators that we detected in response to different activating stimuli (IgE/Ag, Ionomycin, and LPS). However, further studies on the mechanism of action of levetiracetam in MCs are required to better explain the reduced secretion of mediators that we observed in this study.

In conclusion, we here demonstrate that levetiracetam could be efficaciously repurposed to defeat NEPC and to target MCs activation in the context of their supportive function for prostate adenocarcinoma. Our data pave the way to further investigation on the use of levetiracetam in cancer therapy, also in combination with other agents, and in other pathological contexts associated with MCs hyperactivity such as hypersensitivity, mastocytosis, or autoimmune disorders.

## Data Availability Statement

Publicly available datasets were analyzed in this study. This data can be found here: The following datasets were downloaded from the Gene Expression Omnibus (GEO): GSE69903, GSE29958, and GSE65502. Beltran's dataset was downloaded from cBioPortal website as *Z*-score values.

## Ethics Statement

The studies involving human participants were reviewed and approved by Comitato Etico della Fondazione IRCCS Istituto Nazionale dei Tumori. Written informed consent for participation was not required for this study in accordance with the national legislation and the institutional requirements. The animal study was reviewed and approved by the Italian Ministry of Health and performed in accordance to Italian law (D. lgs 26/2014-approval number 515/2016-PR).

## Author Contributions

EJ, MPC, and BF: conception and design. EJ, RS, BF, PO, IF, LB, VC, and FG: development of methodology. RS, BF, PO, FG, GC, VC, CT, and CE: acquisition of data (provided animals, acquired and managed patients, provided facilities, etc.). EJ, BF, RS, PO, FG, GC, CT, CEP, and MPC: analysis and interpretation of data. EJ, BF, RS, CEP, and MPC: writing, review, and/or revision of the manuscript. EJ and MC: study supervision. All authors contributed to the article and approved the submitted version.

## Conflict of Interest

The authors declare that the research was conducted in the absence of any commercial or financial relationships that could be construed as a potential conflict of interest.
