# Supplementary material for: Repurposing of the Antiepileptic Drug Levetiracetam to Restrain Neuroendocrine Prostate Cancer and Inhibit Mast Cell Support to Adenocarcinoma
Source: Front Immunol. 2021 Mar 2;12:622001. doi: 10.3389/fimmu.2021.622001 (PMC7960782; doi:10.3389/fimmu.2021.622001)
Supplement: Supplementary file 1 [file Image_1.pdf]

Sulsenti, Frossi et al, Supplementary Material

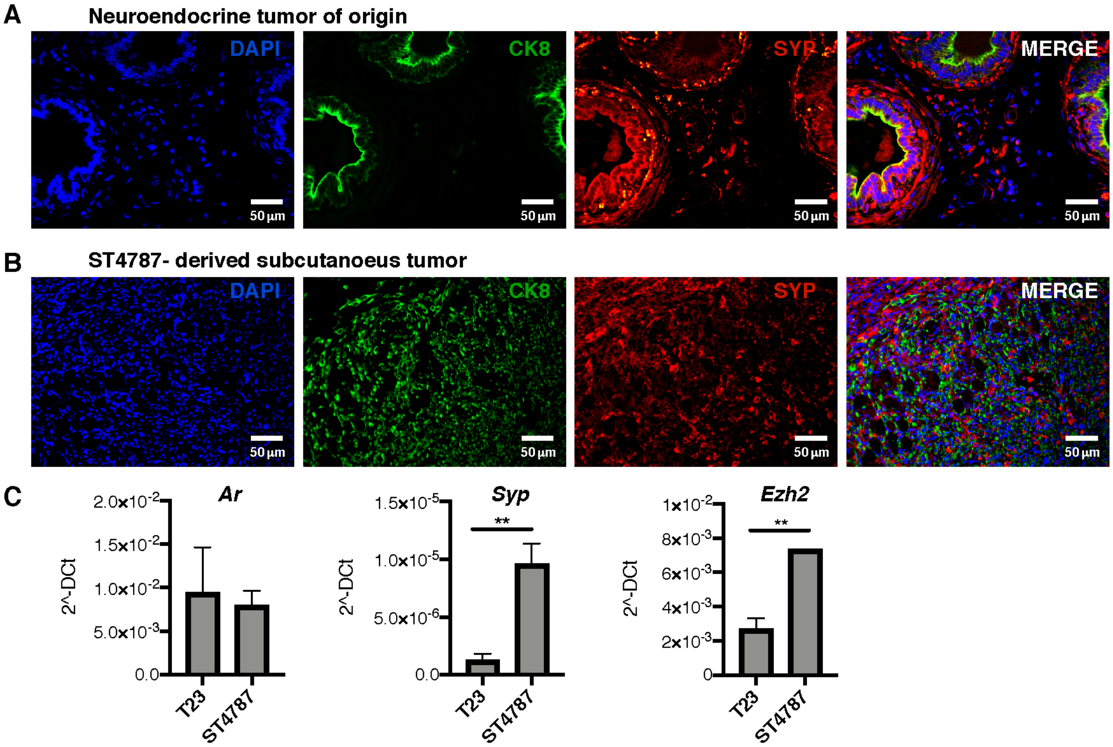

Sulsenti, Frossi et al, Supplementary Figure 1

Supplementary Figure 1. Characterization of the ST4787 NEPC cell line. (A/B) immunofluorescence for the adenocarcinoma marker CK8 (in green) and the NEPC marker synaptophysin (SYP; in red) on the original TRAMP tumor lesion (showing mixed adenocarcinoma and NEPC features) from which the ST4787 cells were derived (A), or in tumor derived from subcutaneous injection of ST4787 cells in syngeneic C57BL/6 mice (B). Blue staining is DAPI. Scale bars indicate magnification. (C) Real time PCR to evaluate transcript levels of Ar and of the NEPC markers Syp and Ezh2 in T23 and ST4787 cells (n=3 per group, experiment was repeated two times). Student's T test ** $p<0.01$.

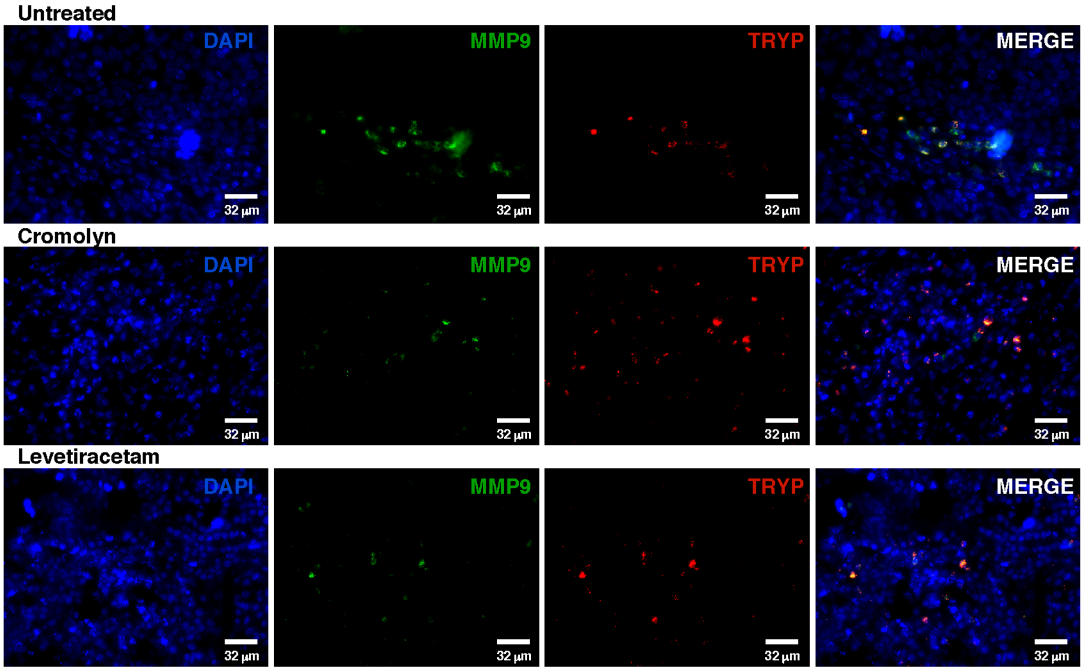

Sulsenti, Frossi et al, Supplementary Figure 2

Supplementary Figure 2. MCs infiltrating T1525 tumors produce MMP9. Immunofluorescence for MMP9 (in green) and the specific MC-marker tryptase (TRYP; in red) on T1525 tumors collected from untreated mice or from mice treated with cromolyn or levetiracetam as in Figure 5A. Blue staining is DAPI. Scale bars indicate magnification. A further digital magnification of these images is reported in Figure 5D.
